# Emission fluxes of styrene monomers and other chemicals for products containing expanded polystyrene beads

**DOI:** 10.1371/journal.pone.0239458

**Published:** 2020-10-01

**Authors:** Atsushi Iizuka, Atsushi Mizukoshi, Miyuki Noguchi, Akihiro Yamasaki

**Affiliations:** 1 Center for Mineral Processing and Metallurgy, Institute of Multidisciplinary Research for Advanced Materials, Tohoku University, Sendai, Miyagi, Japan; 2 Department of Environmental Medicine and Behavioral Science, Kindai University Faculty of Medicine, Osakasayama, Osaka, Japan; 3 Department of Materials and Life Science, Faculty of Science and Technology, Seikei University, Musashino, Tokyo, Japan; Trent University, CANADA

## Abstract

Styrene in indoor air can adversely affect human health. In this study, styrene monomer and other chemical emission fluxes for products containing expanded polystyrene beads (pillows, cushions, and soft toys) were measured at various temperatures to simulate typical product use. The contributions of the products to styrene and other chemical concentrations in indoor air and human exposure to these chemicals were estimated, and health risk assessments were performed. The styrene monomer emission fluxes for the samples at 25°C were between 25.3 and 8.73×10^3^ μg/(m^2^ h). The styrene emission fluxes for the product surfaces increased strongly as the temperature increased, from between 124 and 2.44×10^4^ μg/(m^2^ h) at 36°C (simulating human body temperature) to between 474 and 4.59×10^4^ μg/(m^2^ h) at 50°C (simulating inside an automobile in summer). The hexane, heptane, toluene, octane, ethylbenzene, *m*- and *p*-xylene, *o*-xylene, and dodecane emission fluxes at 25°C for the sample that emitted the analytes most readily were high. The maximum estimated styrene and xylene concentrations in indoor air caused by emissions from expanded polystyrene beads at 36°C in a bedroom and automobile were higher than the relevant guidelines. The maximum contribution of a product containing expanded polystyrene beads in a living room, bedroom, or automobile could cause the total volatile organic compound concentration in air to exceed the advisable value (400 μg/m^3^). The estimated maximum hazard quotients for styrene, toluene, and xylene emitted by a product containing expanded polystyrene beads at 36°C in a bedroom were 0.59, 0.30, and 0.37, respectively. These non-carcinogenic risk values for single products could contribute to the non-carcinogenic risk thresholds being exceeded when multiple products and other sources of chemicals are taken into consideration. The estimated styrene concentrations suggest that products containing expanded polystyrene beads are important sources of styrene to indoor air.

## Introduction

Polystyrene is an amorphous thermoplastic that is extremely fluid, impact resistant, rigid, heat resistant, and cheap. Polystyrene is used widely in packaging, appliances, consumer electronics, the construction industry, the medical industry, and other industries [1]. Polystyrene resin, which is important in industrial processes, contains a high styrene monomer concentration (~1000 mg/kg). Styrene is an important industrial chemical. The estimated compound annual growth rate for worldwide production of styrene between 2017 and 2023 is ~2% per year. It has been predicted that annual global production of styrene by the end of 2023 will be ~33×10^6^ t [2]. Styrene is supplied to indoor air by building materials, consumer products, and tobacco smoke, and styrene concentrations in indoor air between 0.1 and 50 μg/m^3^ have been found. High styrene concentrations may be found in workplaces and home offices because of styrene emissions from laser printers and photocopiers [3]. Styrene may be emitted by reinforced plastic factories, boatbuilding facilities, and polystyrene factories, but humans are likely to be exposed to lower styrene concentrations outdoors than indoors. Styrene concentrations of 0.28–20 μg/m^3^ have been found in outdoor air [3].

Styrene has been classed as posing Group 2A carcinogenic risks (probably carcinogenic) to humans by the International Agency for Research on Cancer [4–6]. It has been found that occupational exposure to styrene may decrease visuomotor accuracy and verbal learning skills [7, 8] and to cause subclinical effects on color vision [9]. The World Health Organization Europe has set air quality guidelines for styrene of 0.26 mg/m^3^ (weekly average) and 70 μg/m^3^ (30 min average; based on the odor threshold) [10]. The Japanese indoor air quality guideline for styrene is 220 μg/m^3^ [11].

Concern about exposure to styrene caused by the use of polystyrene resin led the Japan Styrene Industry Association (JSIA) to measure styrene emission fluxes for polystyrene resin. The styrene emission flux was found to decrease with time after molding and become constant at ~10 d [12]. The JSIA measured styrene emission fluxes for general purpose polystyrene that was very transparent and hard and for high-impact polystyrene containing rubber ingredients. The styrene emission flux for high-impact polystyrene 10 d after injection molding was 3.4 μg/(m^2^ h), which was higher than the emission flux for general purpose polystyrene [12]. The JSIA estimated that the styrene concentration in indoor air in a room (surface area 51 m^2^) covered with polystyrene resin would be 14 μg/m^3^ [12]. This was lower than the Japanese styrene concentration guideline for indoor air of 220 μg/m^3^ [11], so the JSIA concluded that the health effects of styrene released by polystyrene products in such a room would be minor [12].

Polystyrene is often used as expanded polystyrene. Expanded polystyrene beads (EPSBs) are small and have a large specific surface area. EPSBs are used widely in products such as pillows, cushions, and soft toys because of their appealing tactile properties. The styrene emission flux will tend to be higher for EPSBs than polystyrene resin because EPSBs have a large specific surface area and are warmed by the body during use in certain products (e.g., pillows, cushions, and soft toys). There is concern that styrene released from EPSBs used in some products could cause adverse health effects.

Styrene emissions from EPSBs have been determined in only one study. We published a study in 2010 [13] in which we measured styrene and other chemical emission fluxes for the surfaces of three pillows, three cushions, and two soft toys containing EPSBs. The emission fluxes increased markedly with increasing temperature (tests were performed at 25, 36, and 50°C). We concluded that products containing EPSBs can increase styrene concentrations in indoor air, although the styrene concentrations in indoor air that we found (0.70–1.3 μg/m^3^) did not exceed the relevant styrene concentration guideline of 220 μg/m^3^ [13]. Styrene and other chemical emission fluxes are very different for different products that contain EPSBs and for materials of different ages and with different histories, so it is important to determine emission fluxes for various types of sample and compare the new results with the results of the previous study. Products such as pillows and cushions that contain EPSBs are often used in or near the breathing zone, so actual human exposure to a chemical emitted from such products will be higher than estimated from the concentration of the chemical in indoor air caused by emission of the chemical from the product. A more detailed analysis of exposure to chemicals emitted by products containing EPSBs, including estimates of near-field exposure, is therefore required.

The aim of the study presented here was to measure styrene monomer and other chemical emission fluxes for various products (pillows, cushions, and soft toys) that contain EPSBs at different temperatures to simulate typical daily activities and locations. The contributions of the products to styrene and other chemical concentrations in indoor air and human exposure to the chemicals were also estimated, and a health risk assessment was performed.

## Materials and methods

### Materials

Commercial products (four pillows, four cushions, and four soft toys) containing EPSBs were purchased and used for the emission flux measurements. The products were selected because they are typical products containing EPSBs and are often used near the body. Every product was new and not previously used. The products were purchased from physical or online stores over a specific period. The sample characteristics are shown in Table 1. The EPSBs in the samples were all spherical. The surface areas of the EPSBs were estimated from the particle sizes and used to estimate total emissions from the fluxes. It was difficult to find information on the histories and ages of the samples since they had been manufactured. The emission flux measurements for all 12 samples were performed at the same time. The flux measurements were performed within three months of acquiring each sample. The measured chemical fluxes therefore indicated emissions that could occur from commercial products containing EPSBs within three months of purchase.

**Table 1:** Information on the samples of products containing expanded polystyrene beads (EPSBs).

	Sample ID	Bead size [mm]	Sample size [cm]	Estimated surface area [cm^2^]	Material	
					Contents	Outer
Pillow	P-1	~0.7	W35 × L28 × H14	3724	EPSBs	Nylon 85%, PU 15%
	P-2	~1	W24 × L24 × H8	1263	EPSBs	Nylon, PU
	P-3	~0.5	W30 × L16 × H11	1972	EPSBs 95%, PE cotton 5%	Nylon 85%, PU 15%
	P-4	~0.5	W34 × L30 × H11	2280	EPSBs	Double layered: outer: cotton 95%, PU 5% interlock knit, inner: PE 95%, PU 5%
Cushion	C-1	~0.5	φ10–20 × D28 × H24	1810	EPSBs	Nylon 83%, PU 17%
	C-2	~0.7	φ39, inner hole φ11	3454	EPSBs	Nylon 85%, PU 15%
	C-3	~0.7	L50 × 20 × 18	3552	EPSBs	PE 85%, PU 15%
	C-4	~0.5	φ18 × L33	2375	EPSBs	Nylon 80%, PU 20%
Soft toy	T-1	~0.5	W18 × L30 × H18	2205	EPSBs	PE 85%, PU 15%
	T-2	~0.7	W39 × L20 × H22	2222	EPSBs	Nylon 85%, PU 15%
	T-3	~1	φ12	452	EPSBs	Spandex (Nylon 85%, PU 15%)
	T-4	~2–3	φ12 × L27	1244	EPSBs	Cover: cotton 89%, PU 11%, inner cover: PE 92%, PU 8%

ID identification number, P pillow, C cushion, T soft toy.

W width, L length, H height, φ diameter, D depth, PU polyurethane, PE polyester.

### Emission flux measuring device

**Fig 1:**
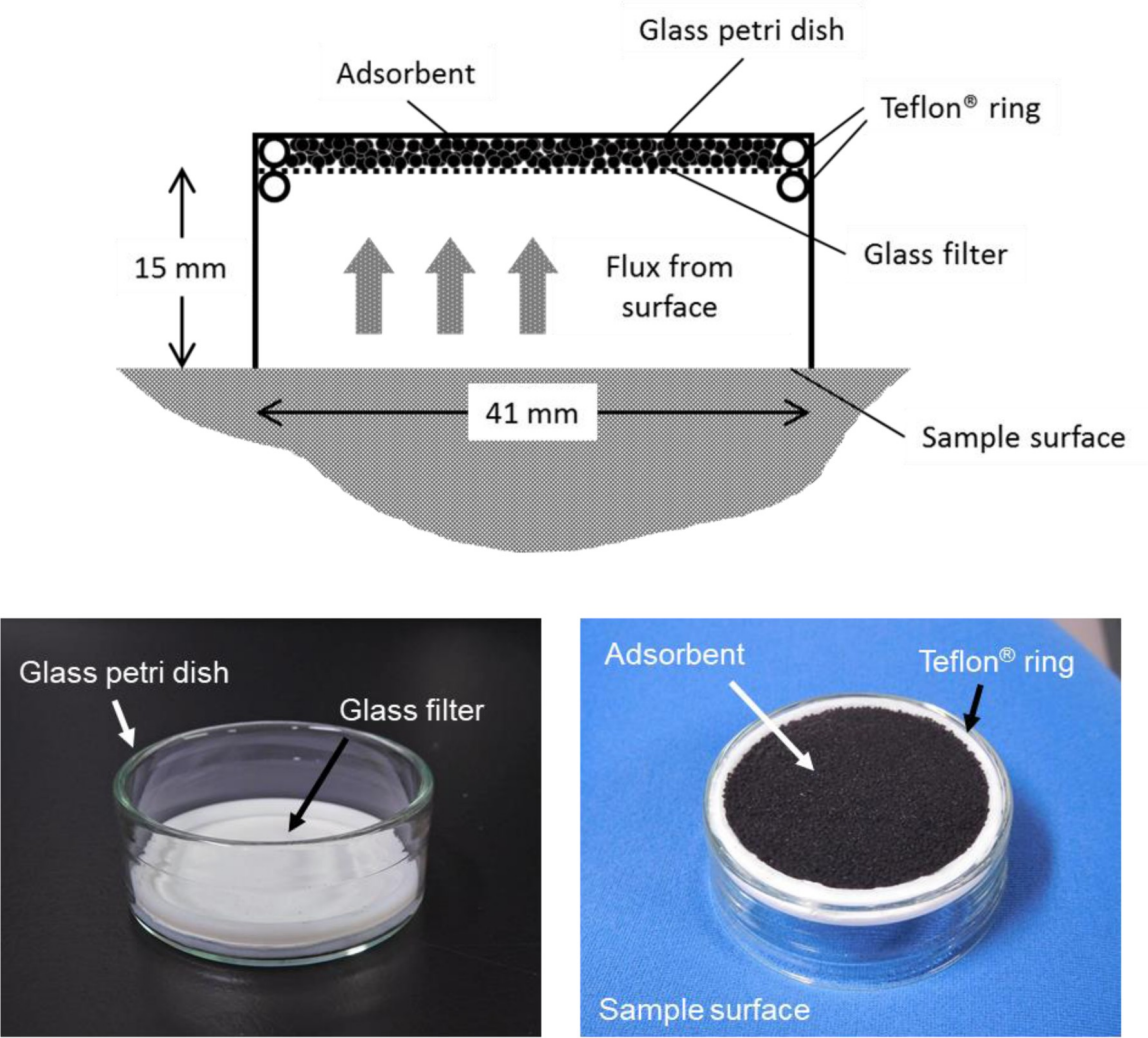
Schematic diagram and photographs of the passive flux sampler.

Passive flux samplers (PFSs) are widely used to measure emission fluxes from material surfaces [13–22]. PFSs were used to measure the chemical fluxes from the surfaces of the samples containing EPSBs. A PFS consisted of an adsorbent on the bottom of a glass Petri dish (inner diameter 41 mm) covered with a glass filter and a Teflon ring (Fig 1). The PFS was placed on the surface of a sample, and chemicals emitted from the sample surface were collected by the adsorbent. The chemicals reached the adsorbent through molecular diffusion following Fick’s Law. Mass transfer in the vicinity of the sample surface will have been dominated by molecular diffusion. The PFS allowed molecular diffusion to occur within the sampler [15]. The PFS diffusion length was set to 15 mm to represent the thickness of gas-phase boundary layers usually found indoors [23]. This meant that the indoor emission flux for a sample could be measured using the PFS. This emission flux measurement method was previously found to give a repeatability, as a relative standard deviation, of 1%–6% for medium density fiberboard [21]. In previous experiments using a reference emission material with the PFS method and a dynamic active sampling headspace method, the emission fluxes determined using the PFS method were less than 20% lower than the emission fluxes determined using the dynamic headspace method [20].

### Emission flux measurement procedure

The emission fluxes of styrene and 42 other chemicals for the surfaces of the samples containing EPSBs were measured using the method described below. The method was a modified version of a method that was described previously [13]. Measurements were made at 25°C (assumed room temperature), 36°C (assumed temperature of a sample placed next to the body), and 50°C (assumed temperature in an automobile in summer).

First, Carbotrap B (360 mg, 20–40 mesh; Merck, Darmstadt, Germany) was weighed and poured into a glass tube (28714-U; inner diameter 6 mm; Merck). The adsorbent was kept in the middle of the glass tube using quartz wool and springs (stainless-steel tension springs for thermal desorption tubes; Merck). The adsorbent was aged by heating it to 300°C for 18 h in a tubular furnace (KTF035N1; Koyo Thermo Systems, Tenri, Japan) with a graphite ferrule (SGI-1/4; GL Sciences, Tokyo, Japan) connected to a 0.25 inch swage fitting to allow nitrogen to be supplied to the adsorbent at a flow rate of 100 mL/min. A glass Petri dish (Fine model 45, outer diameter 46 mm, inner diameter 41 mm; Tokyo Garasu Kikai, Tokyo, Japan) and a glass filter (Whatman GF/C1822-042; GE Healthcare Japan, Tokyo, Japan) trimmed to the same size as the Petri dish were heated to 300°C for 12 h. Teflon O-rings (AS568-029, outer diameter 41.38 mm, and AS568-128, outer diameter 42.94 mm) were heated to 250°C for 12 h in a box furnace (KBF524N1; Koyo Thermo Systems). The parts were assembled as shown in Fig 1 and used as a PFS.

Each sample containing EPSBs was kept in a light-shielding gas-barrier bag filled with nitrogen at room temperature until the flux measurements were made. Each bag was made out of an aluminum/polyethylene terephthalate laminate film (LamisipAL-34L; Seisannipponsha, Tokyo, Japan). The flux measurements were performed within three months of each sample being collected, i.e., each sample was stored in a bag for less than three months. The amounts of the chemicals of interest collected from the inner surfaces of a bag were determined as described below, and the results indicated that negligible amounts of the chemicals of interest were emitted by the samples.

A sample in a bag was placed in a thermostatically controlled chamber (OF-600S; AsOne, Osaka, Japan) for more than 48 h at a specified temperature (25, 36, or 50°C). The temperature inside the chamber was monitored using a sensor (TR-72wf-H; T&D, Matsumoto, Japan). A PFS device was placed on the surface of the sample inside the bag for 1 h to adsorb the chemicals of interest emitted from the sample surface. The sampling time of 1 h was selected to avoid the amounts of chemicals collected by the PFS at 50°C exceeding the upper limits of quantification (LOQs) of the gas chromatography (GC) mass spectrometry (MS) instrument. The same sampling time was used for all of the samples, regardless of the temperature, to maintain comparable sampling conditions. The air in the bag was not allowed to exchange with air outside the bag to maintain the temperature of the sample when the PFS device was placed on the sample. A PFS device was also placed on the inner surface of the bag without a sample for 1 h under the same conditions to check emissions from the bag. The EPSBs were not removed from the samples, and the emission fluxes from the product surfaces were measured directly. Flux measurements for the same samples were performed at the different test temperatures, starting at the lowest temperature.

### Analytical methods

The analytical methods that were used were described in a previous publication [13]. At the end of the sampling period, a known amount of Carbotrap B (180 mg) from the PFS was transferred to a glass tube (2871-U; Merck), then a Teflon storage cap (28019-U; Merck) was attached to each end of the tube. A 20 mg aliquot of Carbotrap B was used for some samples (P-2, P-3, and T-4 at 25 and 36°C and each sample at 50°C) because the amounts of some of the chemicals of interest in 180 mg would have exceeded the upper LOQs of the GC/MS system (typically 500 ng). The glass tube was stored in a light-shielding gas-barrier bag (LamisipAL-15; Seisannipponsha) at room temperature. The chemicals adsorbed to the Carbotrap B in the glass tube were thermally desorbed using a TurboMatrix650 thermal desorption system (PerkinElmer, Waltham, MA, USA). Primary desorption was performed at 300°C for 15 min using helium as the carrier gas at a flow rate of 50 mL/min (inlet split 5 mL/min), and secondary desorption was performed increasing the temperature from 5 to 300°C at 40°C/min using helium as the carrier gas at a flow rate of 10 mL/min (outlet split 9 mL/min). The helium was injected into the GC system (HP6890; Agilent Technologies, Santa Clara, CA, USA) at a flow rate of 1 mL/min (injection ratio 9%). The GC column was an HP-1 (60 m long, 0.25 mm i.d., 1-μm film thickness; Agilent Technologies). The oven temperature program started at 40°C, which was held for 4 min, then increased at 7°C/min to 280°C, which was held for 10 min. The MS system (HP5973N; Agilent Technologies) was used in scanning mode using an m/z ratio range of 33–550. A standard reagent for the analysis of indoor air (4M 9148-U; Merck) containing 50 chemicals each at a concentration of 100 μg/mL in water and methanol was analyzed to allow standard calibration curves to be established. Aliquots of the standard giving 10–1000 ng of each chemical were injected into glass tubes filled with Carbotrap B and analyzed by thermal desorption GC/MS to allow the standard curves to be constructed. The standard curves of 43 chemicals of interest were very linear and had coefficients of determination *R*^2^ >0.97. These 43 chemicals were 2-propanol, methylene chloride, 2-butanone, hexane, chloroform, 1,2-dichloroethane, 2,4-dimethylpentane, 1-butanol, benzene, 1,2-dichloroproapne, bromodichloroethane, trichloroethylene, isooctane, heptane, 4-methyl-2-pentanone, toluene, dibromochloromethane, *n*-butyl acetate, octane, tetrachloroethylene, ethylbenzene, *m*- and *p*-xylene, styrene, *o*-xylene, nonane, α-pinene, 3-ethyltoluene, 4-ethyltoluene, 1,3,5-trimethylbenzene, 2-ethyltoluene, β-pinene, 1,2,4-trimethylbenzene, decane, 1,2,3-trimethylbenene, limonene, undecane, 1,2,4,5-tetramethylbenzene, dodecane, tridecane, tetradecane, pentadecane, and hexadecane. The standard curve ranges for styrene for the samples for which 180 and 20 mg of Carbotrap B were analyzed were 50–500 and 50–1000 ng, respectively. The total volatile organic compound (TVOC) concentrations were calculated using a method that is often used for GC/MS analysis and is described in the ISO 16000–6: 2011 standard [24]. Briefly, the TVOC concentration was calculated as an equivalent concentration of toluene using the total ion abundance for the peaks between the hexane and hexadecane retention times in the GC/MS chromatogram using a calibration curve constructed using the total ion abundances of the toluene peaks in the standards. The lower LOQs, defined as 10 times the signal-to-noise ratios of the chemicals of interest and TVOCs, were mostly 10 ng, but the lower LOQs for 2,4-dimethylpentane and 1,2-dichloropropane were 25 and 15 ng, respectively.

The data presented and used in the calculations were for the samples for which 180 mg of Carbotrap B were used unless the amount of chemical found exceeded the upper LOQ (100 or 500 ng), in which cases the data were for the samples for which 20 mg of Carbotrap B were used, if available. The amount of a chemical adsorbed by the Carbotrap B (360 mg) in the PFS was estimated from the amount of the chemical found in the Carbotrap B in the glass tube (180 or 20 mg) assuming that the amount adsorbed was homogeneously distributed throughout the Carbotrap B in the PFS.

### Emission flux estimation

The emission flux of chemical *i* from the surface of sample *J*_*i*_ (μg/(m^2^ h)) was calculated by dividing the amount of the chemical adsorbed by the product of the sampling time and cross-sectional area of the PFS using the equation

$$J_{i}=\frac{M_{a,i}-M_{b,i}}{t_{a}A_{PFS}},$$ (1)

where *M*_a,*i*_ (μg) is the amount of chemical *i* adsorbed from the sample, *M*_b,*i*_ (μg) is the amount of chemical *i* adsorbed from the inner surface of the bag, *t*_a_ (h) is the sampling time, and *A*_PFS_ (m^2^) is the cross-sectional area of the PFS. When *M*_b,*i*_ (μg) could not be determined by GC/MS, half the *x* intercept of the calibration curve was used. The xylene isomer (*m*, *p*, and *o*) concentrations were added together when the xylene emission flux was calculated.

### Statistical analysis

The relationships between the styrene emission fluxes at 25°C and the maximum bead size and estimated surface area were assessed using Spearman’s rank correlation coefficients. Differences between the styrene emission fluxes at 25°C for the samples with different outer materials (nylon and polyurethane for samples P-1, P-2, P-3, C-1, C-2, C-4, T-2, and T-3 and polyester and polyurethane for samples P-4, C-3, T-1, and T-4) were assessed by performing Mann–Whitney U tests. Differences between the emission fluxes of styrene and three volatile organic compounds (VOCs) (toluene, ethylbenzene, and xylene) and the TVOCs at 25°C found in this study (for which samples were collected and analyzed in 2017) and in a previous study (for which samples were collected and analyzed in 2009) [13] were assessed by performing Mann–Whitney U tests. Statistical analyses were performed using IBM SPSS Statistics Version 22.0 for Microsoft Windows software (IBM, Armonk, NY, USA).

### Estimated chemical concentrations in indoor air and human exposure to chemicals caused by emissions from an EPSB-containing product, and the non-carcinogenic risks posed by the chemicals

The contributions of styrene and three VOCs (toluene, ethylbenzene, and xylene) emitted by EPSB-containing products to the chemical concentrations in indoor air and human exposure to the chemicals were estimated for four cases at different temperatures. Case 1 was a 43.5 m^3^ living room (17.4 m^2^ floor area, 2.5 m high) with an air exchange rate of 0.5 h^−1^ and an exposure time of 6.9 h/d [25] with product temperatures of 25 and 36°C (25°C for room temperature and 36°C for when the sample was in contact with a person). Case 2 was a 27.5 m^3^ bedroom (11.0 m^2^ floor area, 2.5 m high) with an air exchange rate of 0.5 h^−1^ and an exposure time of 8.6 h/d [25] with product temperatures of 25 and 36°C. Case 3 was a cabin (volume 3.89 m^3^) of a stationary automobile with an air exchange rate of 0.55 h^−1^ [26] and an exposure time of 0.33 h/d with product temperatures of 25, 36, and 50°C (50°C was for a closed automobile cabin in summer, and we assumed that a person using the automobile would be exposed to the equivalent of the air in the closed automobile cabin for 0.17 h twice each day). Case 4 was a cabin (volume 3.89 m^3^) of an automobile driving at 60 km/h with an air exchange rate of 11 h^−1^ [26] and an exposure time of 0.9 h/d [27] with product temperatures of 25 and 36°C. The estimated indoor concentrations of chemical *i*, *C*_in,*i*_ (μg/m^3^), for cases 1, 3, and 4 were calculated using the equation

$$C_{in,i}=\frac{J_{i}A_{p}}{EV},$$ (2)

where *J*_*i*_ (μg/(m^2^ h) is the emission flux of chemical *i* from the sample surface calculated using Eq (1), *A*_p_ (m^2^) is the surface area of the EPSB-containing product, *E* (h^−1^) is the air exchange rate, and *V* (m^3^) is the room or cabin volume. Only the contributions of chemicals emitted from the EPSB-containing products to the indoor concentrations were considered. In other words, chemicals in the exterior air and from other indoor sources were not considered. The estimated indoor concentrations therefore only indicate the contributions of the chemicals emitted from the products and are different from the actual indoor concentrations, which also include contributions from the exterior air and other indoor sources.

For case 2, it was assumed that a person would use the EPSB-containing product as a pillow while sleeping in a bedroom, so the distance between the product and the respiration area was smaller than for case 1. The exposure concentration would therefore be higher than was calculated using Eq (2). We therefore used the near-field–far-field model [28], which contains two zones, a zone near and around the source (near field) and the rest of the room (far field). This model has been used to estimate the contributions of emission fluxes from human skin gas to indoor air [29]. If the zones were assumed to be well mixed and air was assumed to exchange between two zones at a specified interzonal flow rate, the estimated near concentration of chemical *i* (near field) could be calculated using the equation

$$C_{in,i}=\frac{J_{i}A_{p}}{EV}+\frac{J_{i}A_{p}}{F_{in}},$$ (3)

where *F*_in_ (m^3^/h) is the interzonal flow rate. The interzonal flow rate was calculated using the equation

$$F_{in}=\frac{1}{2}SAv_{air},$$ (4)

where *SA* (m) is the free surface area of the near field and *v*_air_ (m/h) is the random air speed at the boundary of the near field. The factor 1/2 means that *F*_in_ occurred through air flowing into the near field through one half of the free surface area and through air flowing out through the other half of the free surface area. If the source product was on a flat surface, such as a chair, table, or bed, the near field could be modeled as a hemisphere with diameter *R*_*s*_ (m) around the source product using the equation

$$F_{in}=\frac{1}{2}\left(\frac{1}{2}\pi {R_{S}}^{2}\right)v_{air}.$$ (5)

We assumed *R*_*s*_ = 0.36 m based on a head length of 0.18 m and an air speed *v*_air_ of 0.06 m/s (216 m/h), which is the geometric mean of the wind speeds previously found in 55 indoor workplaces [30].

The estimated average daily exposure to chemical *i*, *E*_exp,*i*_ (μg/d), for each case was then calculated using the equation

$$E_{exp,i}=C_{in,i}t_{exp}IR,$$ (6)

where *t*_exp_ (h/d) is the exposure time per day and *IR* (m^3^/h) is the inhalation rate.

The non-carcinogenic risk posed by chemical *i* for each case was estimated using the hazard quotient (HQ) for that chemical, *HQ*_*i*_ (dimensionless), which was calculated by dividing *E*_exp,*i*_ (μg/m^3^) by the Japanese guideline for the chemical in indoor air, *C*_guide,*i*_ (μg/m^3^), using the equation

$${HQ}_{i}=\frac{E_{exp,i}}{{24C}_{guide,i}IR}.$$ (7)

For styrene, toluene, ethylbenzene, and xylene, the guideline values used were 220, 260, 3800 [11], and 200 μg/m^3^ [31], respectively. Introducing Eq (6) allowed *IR* to be removed and *HQ*_*i*_ to be calculated using the equation

$${HQ}_{i}=\frac{C_{in,i}t_{exp}}{{24C}_{guide,i}}.$$ (8)

No guideline concentration based on health effects is available for TVOCs, so we could not calculate a HQ for TVOCs. The estimated concentration in indoor air was therefore compared with the maximum advisable TVOC concentration of 400 μg/m^3^ [11].

## Results and discussion

The emission fluxes of styrene and 42 other VOCs for the 12 samples at 25°C are shown in Table 2. The emission fluxes for styrene, toluene, ethylbenzene, xylene, and TVOCs, for which VOC emission flux standards for building materials [32] are defined, at different temperatures are shown in Table 3. The results are described in detail and discussed below.

**Table 2:** Emission fluxes (μg/(m^2^ h) for styrene, 42 other volatile organic compounds, and Total Volatile Organic Compounds (TVOC) for the samples (*n* = 12) at 25°C.

	Minimum	Median	Maximum
**Styrene**	**25.3**	**66.5**	**8.73×10**^**3**^
2-Propanol	<14.9	<14.9	<14.9
Methylene chloride	<14.9	<14.9	<14.9
2-Butanone	<14.9	<14.9	<14.9
Hexane	<14.9	**115**	**1.25×10**^**3**^
Chloroform	<14.9	<14.9	**27.6**
1,2-Dichloroethane	<14.9	<14.9	<14.9
2,4-Dimethylpentane	<37.3	<37.3	<37.3
1-Butanol	<14.9	<14.9	**29.1**
Benzene	<14.9	**<15.2**^a^	**37.5**
1,2-Dichloroproapne	<22.4	<22.4	<22.4
Bromodichloroethane	<14.9	<14.9	<14.9
Trichloroethylene	<14.9	<14.9	<14.9
Isooctane	<14.9	<14.9	<14.9
Heptane	<14.9	<15.4^a^	**257**
4-Methyl-2-pentanone	<14.9	<14.9	<14.9
**Toluene**	<14.9	<19.8^a^	**6.22×10**^**3**^
Dibromochloromethane	<14.9	<14.9	<14.9
n-Butyl acetate	<14.9	<14.9	**37.1**
Octane	<14.9	<14.9	**3.11×10**^**2**^
Tetrachloroethylene	<14.9	<14.9	**69.2**
**Ethylbenzene**	**96.3**	**1.03×10**^**3**^	**1.96×10**^**3**^
***m*- and *p*-Xylene**	<14.9	<14.9	**4.07×10**^**3**^
***o*-Xylene**	<14.9	<14.9	**1.35×10**^**3**^
Nonane	<14.9	<14.9	<14.9
α-Pinene	<14.9	<14.9	<14.9
3-Ethyltoluene	<14.9	<14.9	**49.2**
4-Ehtyltoluene	<14.9	<14.9	**23.3**
1,3,5-Trimethylbenzene	<14.9	<14.9	<14.9
2-Ethyltoluene	<14.9	<14.9	<14.9
β-Pinene	<14.9	<14.9	<14.9
1,2,4-Trimethylbenzene	<14.9	<14.9	<14.9
Decane	<14.9	<14.9	**23.9**
1,2,3-Trimethylbenene	<14.9	<14.9	<14.9
Limonene	<14.9	<14.9	<14.9
Undecane	<14.9	<14.9	**20.2**
1,2,4,5-Tetramethylbenzene	<14.9	<14.9	<14.9
Dodecane	<14.9	**98.5**	**166**
Tridecane	<14.9	<14.9	**37.3**
Tetradecane	<14.9	<14.9	**25.6**
Pentadecane	<14.9	<14.9	<14.9
Hexadecane	<14.9	<14.9	<14.9
TVOC	<14.9	**6.40×10**^**2**^	**9.51×10**^**4**^

^a^ One of the median values was higher than the lower limit of quantification (LOQ).

Values higher than the lower LOQ are in bold.

**Table 3:** Emission fluxes for styrene, toluene, ethylbenzene, xylene, and Total Volatile Organic Compounds (TVOC) for the samples at different temperatures.

	Sample ID	Emission flux [μg/m^2^/h]														
		Styrene			Toluene			Ethylbenzene			Xylene			TVOC		
		25°C	36°C	50°C	25°C	36°C	50°C	25°C	36°C	50°C	25°C	36°C	50°C	25°C	36°C	50°C
Pillow	P-1	**82.2**	**201**	**528**	<14.9	<14.9	**149**	**1.25×10**^**3**^	**2.17×10**^**3**^	**7.23×10**^**3**^	<29.9	<29.9	<29.9	**1.22×10**^**3**^	**8.63×10**^**3**^	**7.87×10**^**4**^
	P-2	**7.79×10**^**3**^	**2.12×10**^**4**^	**4.59×10**^**4**^	**5.85×10**^**3**^	**1.46×10**^**4**^	**2.89×10**^**4**^	**1.40×10**^**3**^	**3.63×10**^**3**^	**1.38×10**^**4**^	**2.33×10**^**3**^	**5.77×10**^**3**^	**2.31×10**^**4**^	**9.07×10**^**4**^	**1.26×10**^**5**^	**2.75×10**^**5**^
	P-3	**337**	**1.11×10**^**3**^	**2.33×10**^**3**^	**27.1**	**76.4**	**230**	**1.06×10**^**3**^	**2.30×10**^**3**^	**7.62×10**^**3**^	**167**	**366**	**1.19×10**^**3**^	**7.99×10**^**4**^	**8.88×10**^**4**^	**1.02×10**^**5**^
	P-4	**65.9**	**209**	**1.22×10**^**3**^	<14.9	**28.7**	**209**	**9.59×10**^**2**^	**2.05×10**^**3**^	**8.59×10**^**3**^	<29.9	<29.9	**206**	**690**	**5.27×10**^**3**^	**7.97×10**^**4**^
Cushion	C-1	**93.7**	**351**	**1.52×10**^**3**^	<14.9	**63.6**	**218**	**1.46×10**^**3**^	**3.51×10**^**3**^	**1.44×10**^**4**^	<29.9	<29.9	**315**	**2.62×10**^**3**^	**1.14×10**^**4**^	**9.58×10**^**4**^
	C-2	**25.3**	**124**	**474**	<14.9	<14.9	**128**	**287**	**757**	**3.81×10**^**3**^	<29.9	<29.9	<29.9	**590**	**5.84×10**^**3**^	**7.24×10**^**4**^
	C-3	**67.2**	**211**	**1.23×10**^**3**^	**24.6**	**52.2**	**323**	**1.06×10**^**3**^	**2.13×10**^**3**^	**9.59×10**^**3**^	<29.9	<29.9	**290**	**587**	**3.42×10**^**3**^	**7.20×10**^**4**^
	C-4	**34.0**	**150**	**812**	**530**	**1.25×10**^**3**^	**6.05×10**^**3**^	**96.3**	**257**	**1.76×10**^**3**^	<29.9	<29.9	**170**	<14.9	**1.51×10**^**3**^	**8.14×10**^**4**^
Soft toy	T-1	**43.7**	**134**	**1.28×10**^**3**^	<14.9	**58.8**	**154**	**838**	**1.74×10**^**3**^	**8.28×10**^**3**^	<29.9	<29.9	**196**	<14.9	**3.49×10**^**3**^	**7.95×10**^**4**^
	T-2	**64.4**	**234**	**1.24×10**^**3**^	<14.9	<14.9	**58.1**	**1.01×10**^**3**^	**2.20×10**^**3**^	**8.69×10**^**3**^	<29.9	<29.9	**254**	<14.9	**5.66×10**^**3**^	**7.24×10**^**4**^
	T-3	**65.5**	**226**	**1.62×10**^**3**^	**41.0**	**97.6**	**544**	**350**	**789**	**4.11×10**^**3**^	<29.9	**219**	**1.37×10**^**3**^	<14.9	**4.21×10**^**3**^	**7.58×10**^**4**^
	T-4	**8.73×10**^**3**^	**2.44×10**^**4**^	**4.42×10**^**4**^	**6.22×10**^**3**^	**1.37×10**^**4**^	**2.56×10**^**4**^	**1.96×10**^**3**^	**4.80×10**^**3**^	**1.52×10**^**4**^	**5.42×10**^**3**^	**1.39×10**^**4**^	**4.33×10**^**4**^	**9.51×10**^**4**^	**1.41×10**^**5**^	**2.93×10**^**5**^

Values higher than the lower limits of quantification are in bold.

### Styrene emission fluxes from the product surfaces at 25°C

It can be seen from Table 3 that the styrene emission fluxes at 25°C (room temperature) were between 65.9 and 7.79×10^3^ μg/(m^2^ h) for the four pillows, between 25.3 and 93.7 μg/(m^2^ h) for the four cushions, and between 43.7 and 8.73×10^3^ μg/(m^2^ h) for the four soft toys. The surface areas used to calculate the fluxes were not the EPSB particle surface areas but the product surface areas. Some samples had high styrene fluxes considering that the highest styrene emission flux at 25°C previously found for a product containing EPSBs was 220 μg/(m^2^ h) [13]. The styrene emission fluxes were much higher than the styrene emission flux of 3.4 μg/(m^2^ h) for high-impact polystyrene found by the JSIA [12]. It is difficult to directly compare the fluxes, but the fluxes we found were higher than the standard styrene emission flux for building materials of 32 μg/(m^2^ h) [32]. It should be noted that the total amounts of styrene monomers in the products were not very large, so the styrene emission flux would have decreased over time. It is important for temporal variations in the chemical fluxes for products containing EPSBs over long periods to be determined in a future study.

The Spearman’s rank correlation coefficients indicated that the relationships between the styrene emission flux and the maximum bead size (p = 0.29) and estimated surface area (*p* = 0.145) were not statistically significant (i.e., *p*>0.05). The Mann–Whitney *U* test results indicated that the differences between the styrene emission fluxes for the different outer materials were not statistically significantly different (*p* = 0.81).

### Temperature dependence of the styrene emission flux

The styrene emission fluxes for the product surfaces increased as the temperature increased. The styrene emission fluxes were between 124 and 2.44×10^4^ μg/(m^2^ h) at 36°C and between 474 and 4.59×10^4^ μg/(m^2^ h) at 50°C. The styrene emission flux increased consistently as the temperature increased, but the absolute amounts of styrene emitted varied widely.

Emission fluxes were measured at only three temperatures, but we created an Arrhenius plot of the styrene fluxes at the different temperatures and estimated that the apparent activation energies for styrene emission were 51.4–109 kJ/mol. The activation energy for the thermal-degradation reaction of polystyrene resin has been found to be 210 kJ/mol [33]. We concluded that the rate-limiting step for styrene emission was therefore mass transfer of styrene, such as transfer within the EPSBs, transfer through the empty spaces inside the products, or transfer within the outer materials of the products. The apparent activation energies for ethylbenzene emission were 56.5–93.6 kJ/mol. The activation energies for the other chemicals could not be well estimated because many of the data were below the detection limits (at least three values were required to estimate the activation energy).

### Emission fluxes of other chemicals at 25°C

As shown in Table 3, styrene emissions were measured for all of the samples. The emission fluxes of hexane, heptane, toluene, octane, ethylbenzene, *m*- and *p*-xylene, *o*-xylene, and dodecane were also high. Toluene, xylene, and other VOCs are used as additives in expanded resins. Styrene monomers are generally produced from ethylbenzene, and it has been found that commercial styrene monomers contain ethylbenzene, *t*-butylcatechol, aldehydes, and benzene as impurities [34]. These chemicals can be found as impurities in styrene polymers and could be emitted from EPSBs.

### Comparison with emission data published in 2010

**Fig 2:**
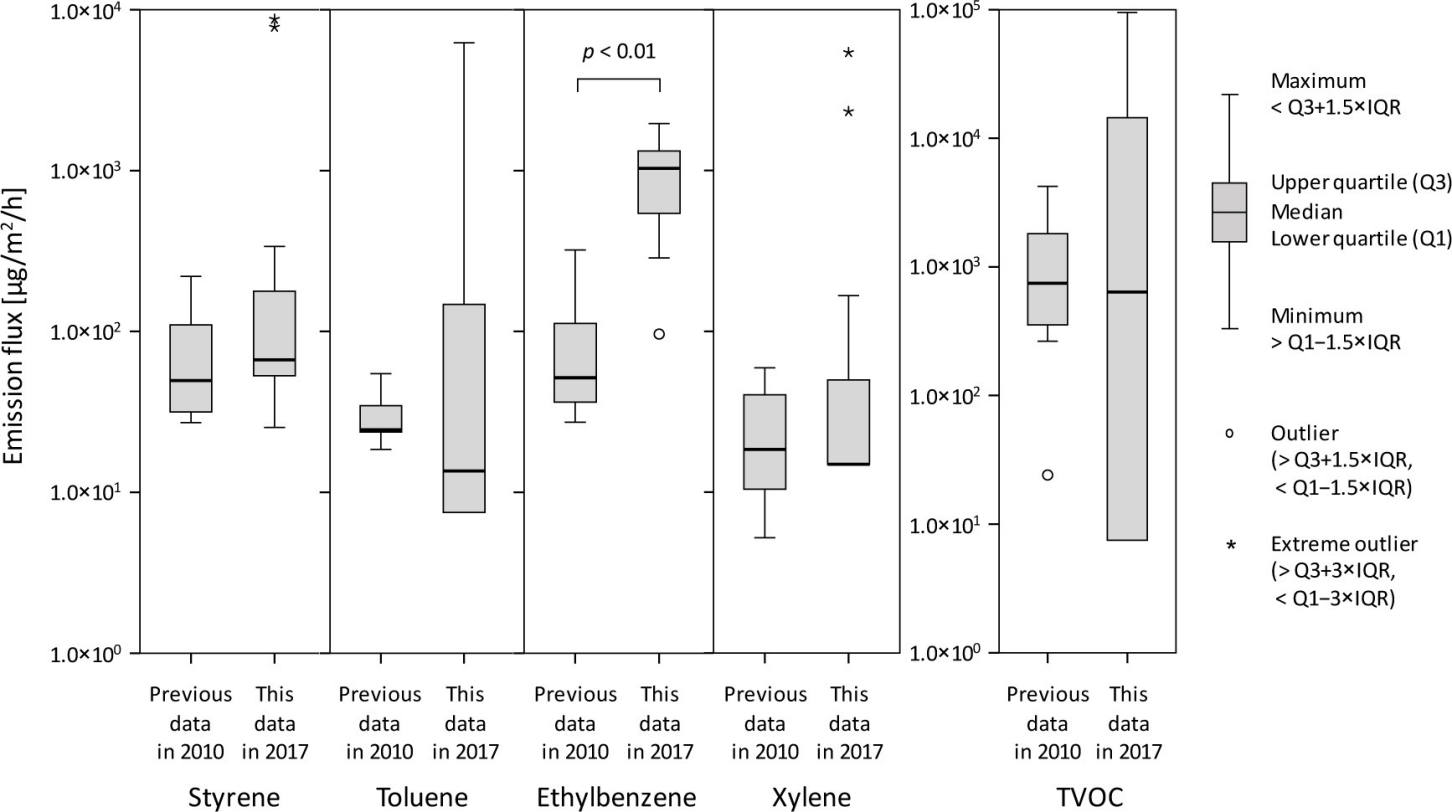
Styrene, toluene, ethylbenzene, xylene, and Total Volatile Organic Compound (TVOC) emission fluxes at 25°C found in this study (performed in 2017) and in a previous study. IQR means the interquartile range.

The measured chemical emission fluxes for the 12 samples at 25°C and for eight commercial products at 25°C found in a previous study [13] were compared. The emission fluxes are shown in Fig 2. Note that HQs for a living room, bedroom, and automobile cabin were not compared. The styrene, toluene, xylene, and TVOC emission fluxes found in the different studies were not significantly different. However, the ethylbenzene emission fluxes found in our study and the previous study were significantly different (Mann–Whitney *U* test, *p*<0.01). The ethylbenzene emission fluxes found in our study (performed in 2017) were higher than the emission fluxes found in the previous study (published in 2010). Ethylbenzene is the main starting chemical when styrene monomers are synthesized [6] and can be present in the styrene monomer product as an impurity. The ethylbenzene remaining the styrene can be transferred to EPSBs produced from the styrene and be emitted from the EPSBs. These results may indicate that the amounts of ethylbenzene in styrene monomers have recently decreased. There could be a number of reasons for this, such as a different synthesis pathway being used or impurities in styrene being removed before EPSBs are produced.

### Estimated concentrations of chemicals in indoor air caused by emissions from EPSB-containing products

The estimated contributions of emissions from the EPSB-containing products to the styrene, toluene, ethylbenzene, xylene, and TVOC concentrations in indoor air and the HQs for styrene, toluene, ethylbenzene, and xylene for the four cases are shown in Table 4. For a living room (case 1) at 25 and 36°C, the median and maximum indoor styrene, toluene, ethylbenzene, and xylene concentrations were lower than the guideline concentrations for indoor air and the HQs were all <1. However, the maximum TVOC concentration was higher than the maximum advisable concentration of 400 μg/m^3^ [11]. For a bedroom (case 2), the estimated indoor concentrations were 2.6 times higher than the estimated indoor concentrations for case 1 because the near-field concentrations were used and the room volume was lower. For case 2, the maximum styrene and xylene concentrations at 36°C were higher than the relevant guidelines and the maximum TVOC concentrations at 25 and 36°C were higher than the maximum advisable concentration. The HQs were <1, meaning that one EPSB-containing product would not pose a non-carcinogenic risk, but the HQs for styrene, toluene, and xylene were relatively high (0.59, 0.30, and 0.37, respectively) at 36°C, meaning that emissions of these chemicals from EPSBs may contribute to the non-carcinogenic risk thresholds for these chemicals being exceeded when other sources of styrene or multiple EPSB-containing products are present. For a stationary automobile cabin (case 3), the median TVOC concentrations in indoor air at 36 and 50°C were higher than the maximum advisory concentration and the maximum styrene, toluene, xylene, and TVOC concentrations were higher than the relevant guidelines. However, all of the HQs were <1 because the exposure time was short. For a moving automobile cabin (case 4), the concentrations of all of the chemicals were lower than the regulatory values and the HQs were <1. As mentioned above, the emission fluxes found in this study will be different from emission fluxes in real buildings because of differences in the thicknesses of the boundary layers next to sample surfaces [18], sample thicknesses, ventilation rates, temperatures, and other parameters. The HQs we calculated may therefore not be applicable to real buildings and vehicle cabins. The difference between the HQs determined using the PFS method and dynamic headspace method could have been 20% [20]. The highest HQ for case 2 at 36°C determined using the PFS method was 0.59. The highest HQ for case 2 at 36°C determined using the dynamic headspace method would therefore be 0.74, which is close to 1, although other parameters could cause this to be an overestimate or underestimate (see the summary of the limitations of the research section).

**Table 4:** Estimated contributions of emissions from products containing expanded polystyrene beads to the concentrations of styrene, toluene, ethylbenzene, xylene and Total Volatile Organic Compounds (TVOC) in indoor air and the Hazard Quotients (HQs) for four cases.

		Styrene			Toluene			Ethylbenzene			Xylene			TVOC		
		25°C	36°C	50°C	25°C	36°C	50°C	25°C	36°C	50°C	25°C	36°C	50°C	25°C	36°C	50°C
Case 1: Living room^a^																
Estimated indoor concentration [μg/m^3^]	Median	7.35×10^−1^	2.66	-	<2.41×10^−1^	<5.62×10^−1^	-	9.81	21.3	-	<4.00×10^−1^	<4.65×10^−1^	-	9.47	75.3	-
	Maximum	50.0	140	-	35.6	84.8	-	21.5	37.1	-	31.0	79.5	-	**725**	**806**	-
HQ	Median	9.60×10^−4^	3.48×10^−3^	-	<2.67×10^−4^	<6.22×10^−4^	-	7.43×10^−4^	1.61×10^−3^	-	<5.75×10^−4^	<6.68×10^−4^	-	-	-	-
	Maximum	6.53×10^−2^	1.83×10^−1^	-	3.94×10^−2^	9.38×10^−2^	-	1.63×10^−3^	2.81×10^−3^	-	4.46×10^−2^	1.14×10^−1^	-	-	-	-
Case 2: Bedroom^b^																
Estimated indoor concentration [μg/m^3^]	Median	1.89	6.84	-	<6.21×10^−1^	<1.45	-	25.2	54.7	-	<1.03	<1.19	-	24.4	194	-
	Maximum	128	**359**	-	91.5	218	-	55.2	95.4	-	79.7	**204**	-	**1.86×10**^**3**^	**2.07×10**^**3**^	-
HQ	Median	3.08×10^−3^	1.11×10^−2^	-	<8.55×10^−4^	<1.99×10^−3^	-	2.38×10^−3^	5.16×10^−3^	-	<1.84×10^−3^	<2.14×10^−3^	-	-	-	-
	Maximum	2.09×10^−1^	**5.86×10**^**−**1^	-	1.26×10^−1^	**3.01×10**^**−1**^	-	5.21×10^−3^	9.00×10^−3^	-	1.43×10^−1^	**3.66×10**^**−1**^	-	-	-	-
Case 3: Automobile cabin when stationary^c^																
Estimated indoor concentration [μg/m^3^]	Median	7.47	27.0	129	<2.45	<5.72	21.7	99.8	216	868	<4.07	<4.47	26.5	96.3	**766**	**9.21×10**^**3**^
	Maximum	**508**	**1.42×10**^**3**^	**2.71×10**^**3**^	**362**	**862**	**1.71×10**^**3**^	218	377	1.59×10^3^	**315**	**808**	**2.52×10**^**3**^	**7.37×10**^**3**^	**8.19×10**^**3**^	**1.70×10**^**4**^
HQ	Median	4.67×10^−4^	1.69×10^−3^	8.08×10^−3^	<1.30×10^−4^	<3.02×10^−4^	1.15×10^−3^	3.61×10^−4^	7.83×10^−4^	3.14×10^−3^	<2.80×10^−4^	<3.25×10^−4^	1.82×10^−3^	-	-	-
	Maximum	3.17×10^−2^	8.88×10^−2^	1.69×10^−1^	1.91×10^−2^	4.56×10^−2^	9.02×10^−2^	7.90×10^−4^	1.37×10^−3^	5.76×10^−3^	2.17×10^−2^	5.56×10^−2^	1.73×10^−1^	-	-	-
Case 4: Automobile cabin when driving^d^																
Estimated indoor concentration [μg/m^3^]	Median	3.74×10^−1^	1.35	-	<1.23×10^−1^	<2.86×10^−1^	-	4.99	10.8	43.4	<2.03×10^−1^	<2.36×10^−1^	-	4.82	38.3	-
	Maximum	25.4	71.1	-	18.1	43.1	-	10.9	18.9	79.6	15.8	40.4	-	368	**410**	-
HQ	Median	6.37×10^−5^	2.30×10^−4^	-	<1.77×10^−5^	<4.12×10^−5^	-	4.92×10^−5^	1.07×10^−4^	4.28×10^−4^	<3.81×10^−5^	<4.43×10^−5^	-	-	-	-
	Maximum	4.33×10^−3^	1.21×10^−2^	-	2.61×10^−3^	6.22×10^−3^	-	1.08×10^−4^	1.86×10^−4^	7.85×10^−4^	2.95×10^−3^	7.58×10^−3^	-	-	-	-

^a^ Room volume 43.5 m^3^, air exchange rate 0.5 h^−1^, exposure time 6.9 h/d.

^b^ Room volume 27.5 m^3^, air exchange rate 0.5 h^−1^, exposure time 8.6 h/d.

^c^ Cabin volume 3.89 m^3^, air exchange rate 0.55 h^−1^, exposure time 0.33 h/d.

^d^ Cabin volume 3.89 m^3^, air exchange rate 11 h^−1^, exposure time 0.9 h/d.

- Not calculated because an indoor temperature of 50°C was not used in cases 1 and 3 and there is no indoor TVOC concentration guideline.

Estimated indoor concentrations higher than the relevant guidelines or advisable value and HQs >0.3 are shown in bold type.

The odor detection threshold for styrene has been found to be 70 μg/m^3^. The air quality guideline could be based on the odor threshold, i.e., the maximum 30 min average styrene concentration in air should be lower than 70 μg/m^3^ [10]. The estimated styrene concentrations in indoor air were compared with the air quality guideline for styrene (70 μg/m^3^) [10]. The maximum styrene concentrations for case 1 at 36°C and case 2 at 25 and 36°C were higher than the air quality guideline for styrene. It was difficult to directly compare the results because the assumed exposure periods for cases 3 and 4 were <30 min, the median concentration for case 3 at 50°C, the maximum concentrations for case 3 at 25, 36, and 50°C, and the maximum concentration for case 4 at 36°C were also higher than the air quality guideline for styrene. This means that styrene would be able to be perceived as an odor in these cases.

In a nationwide survey of indoor air in summer 2013 [35], the median and maximum styrene concentrations were 1.1 and 66 μg/m^3^, respectively, in living rooms and 1.2 and 110 μg/m^3^, respectively, in bedrooms. These concentrations were comparable to the estimated concentrations found in our study (the median and maximum concentrations in indoor air were 7.35×10^−1^ and 50.0 μg/m^3^, respectively, in a living room (case 1) at 25°C and 1.89 and 128 μg/m^3^, respectively, in a bedroom (case 2) at 25°C). In a survey of indoor air performed between 2009 and 2011 [36], the arithmetic mean styrene concentration in Canadian homes was 1.13 μg/m^3^ and the geometric mean concentrations in houses and apartments in which people smoked were significantly higher (by 0.44 and 0.70 μg/m^3^, respectively) than the geometric mean concentrations in houses and apartments in which people did not smoke. In our study, the median estimated increase in the styrene concentration caused by the presence of an EPSB-containing product was 1.89 μg/m^3^ at 25°C, which was comparable to the increase found to be caused by smoking in the study of Canadian homes. This suggests that EPSB-containing products could be sources of styrene to indoor air. There are other sources of styrene to indoor air, including floor coverings [37], cigarette butts [38], and 3D printers [39, 40]. These sources will cumulatively increase the styrene concentration in indoor air. Appropriate methods for controlling sources of styrene and ventilation are therefore important to decrease human exposure to styrene and other chemicals.

### Summary of the limitations of the study

The limitations of the study are summarized here. First, the number of products used to represent common styrene-emitting products was small (12). It was difficult to determine the dates the EPSBs used in the 12 products were manufactured. It took a certain amount of time to acquire all 12 products, but the flux measurements were performed within three months of the first product being collected. The storage conditions (e.g., exposed to air or sealed) before each product was purchased may have affected the emission flux, and different emission fluxes may have been found even for the same product stored under different conditions. The chemical emission fluxes we found should therefore be viewed as possible emission fluxes for commercial products containing EPSBs within three months of purchase. The emission flux for a product was calculated using only time-averaged emissions at one point on the product surface (without duplication). The emission flux could vary over time and position on the product surface. The sample characteristics (e.g., shape and size) could have affected the emission measurements. The two-zone model used in the study was a simplified model. More detailed analysis using the computational fluid dynamics method should be performed. The styrene and other chemicals that were emitted could have reacted with other chemicals (e.g., hydroxyl radicals and ozone) to produce gaseous byproducts such as formaldehyde and benzaldehyde [41] and secondary organic particles [42, 43]. The calculated pseudo-first order rate constant for the reaction between styrene and ozone (20 ppb) has been found to be 0.03 h^−1^ [44], which corresponded to 6% of the air exchange rate of 0.5 h^−1^ in case 1 (living room) and case 2 (bedroom). The effects of ozone and other chemicals on the measured emission fluxes and concentrations in indoor air were not considered in our study. Despite the limitations mentioned above, we believe that the results provide useful insights into emissions of chemicals by commercial products containing EPSBs. More comprehensive studies of styrene and other chemical fluxes from EPSB-containing products, including long-term temporal variations, should be performed in future.

## Conclusions

Styrene monomer and other chemical emission fluxes for products containing EPSBs were measured using PFSs. The styrene monomer emission fluxes for all of the samples at 25°C were between 25.3 and 8.73×10^3^ μg/(m^2^ h). Increasing the temperature from 25 to 36°C (the temperature a product may reach when next to the body) or 50°C (the temperature a product may reach in an automobile cabin in summer) greatly increased the styrene emission fluxes from the product surfaces. The hexane, heptane, toluene, octane, ethylbenzene, *m*- and *p*-xylene, *o*-xylene, and dodecane emission fluxes were also large. The maximum estimated contributions of styrene and xylene emitted by products containing EPSBs to the styrene and xylene concentrations in indoor air in a bedroom at 36°C were higher than the relevant guidelines. The maximum styrene, toluene, and xylene HQs at 36°C in a bedroom were 0.59, 0.30, and 0.37, respectively, meaning that one product would not pose a non-carcinogenic risk. However, EPSB-containing products may contribute to the non-carcinogenic risks posed by these chemicals exceeding the relevant thresholds because other sources of styrene and other chemicals and multiple EPSB-containing products may be present in a room. The estimated styrene concentrations in indoor air suggest that EPSB-containing products may be sources of styrene to indoor air.
